# Seasonal immunoregulation in a naturally-occurring vertebrate

**DOI:** 10.1186/s12864-016-2701-7

**Published:** 2016-05-18

**Authors:** Martha Brown, Pascal Hablützel, Ida M. Friberg, Anna G. Thomason, Alexander Stewart, Justin A. Pachebat, Joseph A. Jackson

**Affiliations:** IBERS, Aberystwyth University, Aberystwyth, SY23 3DA UK; School of Environment and Life sciences, University of Salford, Salford, M5 4WT UK; Cardiff School of Biosciences, University of Cardiff, Cardiff, CF10 3AX UK

**Keywords:** Seasonality, RNAseq, Teleost, Three-spined stickleback, Immunity, Immunoregulation, Ecoimmunology, Wildlife

## Abstract

**Background:**

Fishes show seasonal patterns of immunity, but such phenomena are imperfectly understood in vertebrates generally, even in humans and mice. As these seasonal patterns may link to infectious disease risk and individual condition, the nature of their control has real practical implications. Here we characterize seasonal dynamics in the expression of conserved vertebrate immunity genes in a naturally-occurring piscine model, the three-spined stickleback.

**Results:**

We made genome-wide measurements (RNAseq) of whole-fish mRNA pools (*n* = 36) at the end of summer and winter in contrasting habitats (riverine and lacustrine) and focussed on common trends to filter habitat-specific from overarching temporal responses. We corroborated this analysis with targeted year-round whole-fish gene expression (Q-PCR) studies in a different year (*n* = 478). We also considered seasonal tissue-specific expression (6 tissues) (*n* = 15) at a third contrasting (euryhaline) locality by Q-PCR, further validating the generality of the patterns seen in whole fish analyses. Extremes of season were the dominant predictor of immune expression (compared to sex, ontogeny or habitat). Signatures of adaptive immunity were elevated in late summer. In contrast, late winter was accompanied by signatures of innate immunity (including IL-1 signalling and non-classical complement activity) and modulated toll-like receptor signalling. Negative regulators of T-cell activity were prominent amongst winter-biased genes, suggesting that adaptive immunity is actively down-regulated during winter rather than passively tracking ambient temperature. Network analyses identified a small set of immune genes that might lie close to a regulatory axis. These genes acted as hubs linking summer-biased adaptive pathways, winter-biased innate pathways and other organismal processes, including growth, metabolic dynamics and responses to stress and temperature. Seasonal change was most pronounced in the gill, which contains a considerable concentration of T-cell activity in the stickleback.

**Conclusions:**

Our results suggest major and predictable seasonal re-adjustments of immunity. Further consideration should be given to the effects of such responses in seasonally-occurring disease.

**Electronic supplementary material:**

The online version of this article (doi:10.1186/s12864-016-2701-7) contains supplementary material, which is available to authorized users.

## Background

Seasonal immune function has often been observed in vertebrates [1], including humans [2, 3], but is relatively poorly understood. As with more studied circadian rhythms, though, there are fundamental implications for health [4, 5] (e.g., effects on vaccination and diseases linked to immune function). Taking a comparative approach [6] and considering the conserved genes of the vertebrate immune system, here we use transcriptomic measurements to reveal seasonal re-adjustments of immunity in a naturally-occurring teleost model. Crucially, through focussing on wild organisms exposed to real-world environmental extremes, we expected to discover more measurable variation than would be the case in a study of domesticated animals, where seasonal variation may be muted by anthropogenic influences.

We chose the three-spined stickleback (*Gasterosteus aculeatus*) as a subject because it has an annotated whole genome sequence [7] and occurs accessibly in highly seasonal natural habitats. Also, a considerable knowledge base exists for this species: it is a highly studied model organism [8, 9], and there are particularly detailed ecological studies relating to our main study area, mid Wales [10, 11]. We compared the transcriptomes of populations in late winter and late summer (outside of the breeding season, to reduce complexity) in ecologically divergent natural populations, reasoning that a focus on common responses would provide a way to filter overriding seasonal trends from locality-specific variation. We chose to primarily use global mRNA extracts from individual whole fishes rather than from isolated cell populations or tissues. This was because a fully reductionist approach to cell populations would be impractical, and because the majority of the teleost immune system is likely to be diffusely distributed in the gut, under the skin and mucosal surfaces and in association with the gills and liver (where, for example, complement proteins are mostly synthesized) [12–16].

By considering global (whole-fish) samples, we were thus able to take a holistic view of which immune system pathways are differentially expressed at seasonal extremes. We corroborated our transcriptomic analyses by targeted gene expression measurements in year-round samples of fishes from the original sites in a new annual cycle, and by tissue-specific analyses at a further site. Moreover, using network analyses we were able to ask what genes are important in regulating seasonal immune function and how do seasonally-biased immune networks interact with other seasonally-biased organismal processes?

## Results

### Seasonal expression bias of immune system genes occurs against a well-defined genome-wide seasonal signature

We analyzed the global (whole fish) transcriptomes of *G. aculeatus* from two contrasting habitats in mid Wales, River (Afon) Rheidol (RHD) and Lake (Llyn) Frongoch (FRN), in September 2012 and March 2013. To begin our analysis we considered, genome-wide, which genes were associated with seasonal expression bias. At FRN, 4464 genes were significantly differentially expressed from summer to winter with an individual cut-off (*P* = 0.05) and 1678 with a false discovery rate (FDR)-adjusted cut-off. At RHD, 4383 genes were significantly differentially expressed with an individual cut-off and 2067 with an FDR-adjusted cut-off. Genes that were seasonally differentially expressed at both localities tended, overwhelmingly, to show synchronous expression (in the same direction at the same season across sites).

We hypothesized that these synchronously differentially expressed genes would also be those contributing to overarching seasonal responses. Thus, we categorized such genes on the basis that they were significantly (*P* <0.05) differentially expressed, in the same direction, at both sites at the individual error rate (in practice a more stringent cut-off than an FDR-adjusted *P* = 0.05 for one locality). Following this criterion, 1263 genes were differentially expressed in a consistent direction (Additional file 1: Table S1 shows those with Ensembl annotations), 850 increasing expression during winter (winter-biased) and 413 increasing expression during summer (summer-biased).

**Fig. 1:**
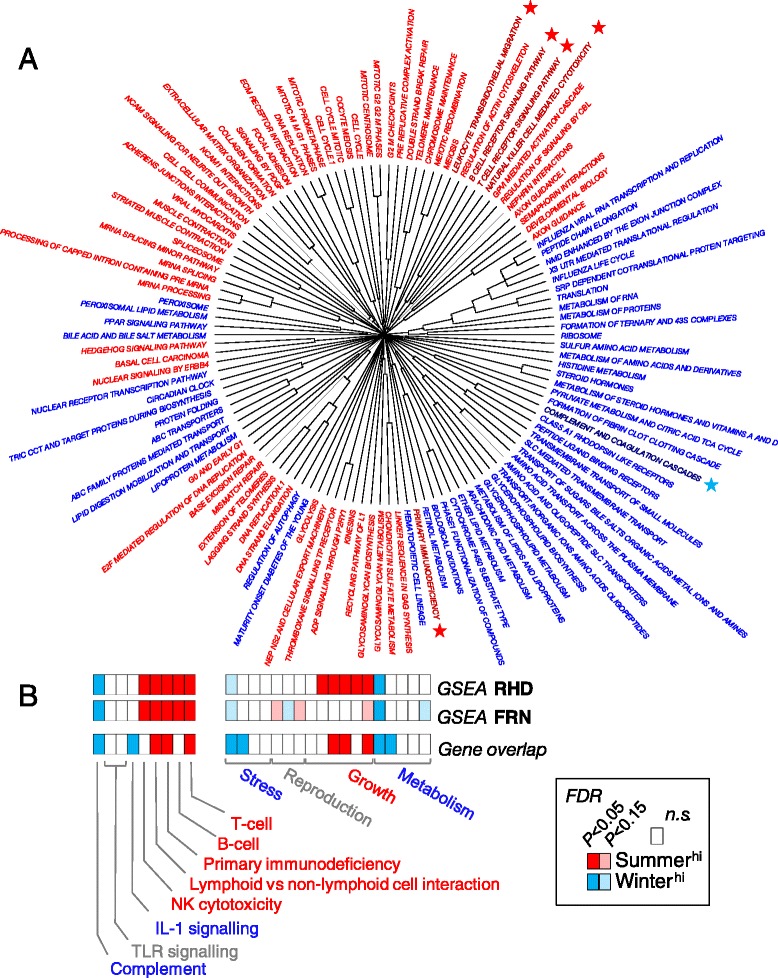
Distinctive immunological and genome-wide gene expression signatures occurred at seasonal extremes. **a** Gene sets with significant summer (red) or winter (blue) expression bias as indicated by gene set enrichment analysis (GSEA). Ranked differential gene expression was compared, separately for the RHD and FRN sites, to global KEGG and REACTOME gene sets, and sets are shown where the combined FDR *P* value was significant (<0.05); gene set names are truncated but shown in full in Additional file 2: Table S2; stars indicate immunologically-relevant gene sets; the central dendrogram indicates the degree of overlap between gene sets. **b** Analyses of selected gene sets (Additional file 3: Table S3) representing immunological pathways and organismal signatures of stress, reproduction, growth and metabolism. Individual colour panels correspond, left to right, to the order of gene sets in Additional file 3: Table S3. These sets were considered by GSEA for RHD and FRN separately, and by gene overlap (hypergeometric distribution) for the overall summer and winter-biased gene sets (defined as those genes having significant expression differences, in the same direction, at both FRN and RHD)

We then considered genome-wide winter-summer expression changes in functional gene sets using gene set enrichment analyses (GSEA) (Fig. 1), focussing on predicted orthologues of human genes (*n* = 11455 genes). We compared ranked gene expression to KEGG and REACTOME pathways, globally (Fig. 1a, Additional file 2: Table S2). We also compared the ranked genes to a selected group of gene sets reflecting different organismal processes and immunological pathways (Fig. 1b, Additional file 3: Table S3). Finally, we considered hypergeometric overlap of the selected gene sets with the summer- and winter-biased genes defined above (Fig. 1b). Taken together, these analyses (Fig. 1) indicated signatures of growth in summer (summer bias of pathways reflecting mitotic activity, ECM processes, neural development, developmental biology) and physiological challenge in winter (winter bias of pathways reflecting autophagy, metabolism, biological oxidation and the transport of a wide range of biomolecules). In all analyses, some immune pathways were seasonally-biased, with innate processes (complement cascade) emphasised in winter and adaptive (lymphocyte) processes in summer (Fig. 1).

The above analyses were carried out on expression data un-adjusted for individual size, as this variable was (intentionally) approximately balanced across winter and summer samples. However, as our sampling points bounded a non-recruiting population ageing in the interval between breeding seasons, we considered in more detail the potential influence of ontogenetic stage. It is likely, given the months (March to September) in which we recorded reproductive activity in the field, and taking into account slower biological ageing at lower temperatures (through measuring age in growing degree days), that the 0^+^ cohort in our approximately size-matched summer and winter samples would have included individuals widely overlapping in effective age (see Additional file 4: Figures S1-S2). Furthermore, as our sampling deliberately selected a wide range of fish sizes, it is probable that 0^+^ and 1^+^ cohorts [11] were represented, resulting in a very extensive overlap of effective ages between summer and winter samples. A close association between body size and age allows age to be partitioned from season in statistical models by the use of a size metric, such as body length, as a surrogate. This is validated by data from experiments in artificial outdoor habitats, where we found that time explains at least 57 % of the variation in length (see Additional file 4: Figure S1). In order to control for age (length) effects we applied general linear models (LMs) to each in turn of the 11455 genes in the GSEA dataset, including main effects for season, length, sex and site. We found that season was the dominant predictor of gene expression (see Additional file 5: Figure S3a). Consistent with this gene-by-gene analysis, a multivariate principal co-ordinates analysis (PCO) of the same data demonstrated marked differentiation across seasons against the two major axes (axis 1, *P* = 0.003; axis 2, *P* = 0.0004), but none for length, sex or site (see Additional file 5: Figure S3b). We also re-ran “global” GSEA analyses (against all KEGG and REACTOME pathways), first with genes ranked by confounder-adjusted seasonal effect, and then with genes ranked by confounder-adjusted length effects (ranking was based on parameter sign and effect size, η^2^, in the LMs above). We found a similar outcome in the analysis ranked by confounder-adjusted seasonal effect to in the unadjusted analysis shown in Fig. 1a, with the two analyses sharing 67 gene sets that were significantly seasonally enriched (FDR-adjusted *P* = 0.05 cut-off), including all of the immunological sets except leucocyte transendothelial migration (Additional file 6: Table S4). In contrast, there was a distinctive outcome in the analysis ranked by confounder-adjusted length effect, where only 8 enriched gene sets were shared with the analysis shown in Fig. 1a, including none of the immunological sets. Thus, the effect of season was a very dominant one, emerging clearly in analyses even without adjustment for ontogeny.

### Season is a dominant and consistent influence on immune gene expression

**Fig. 2:**
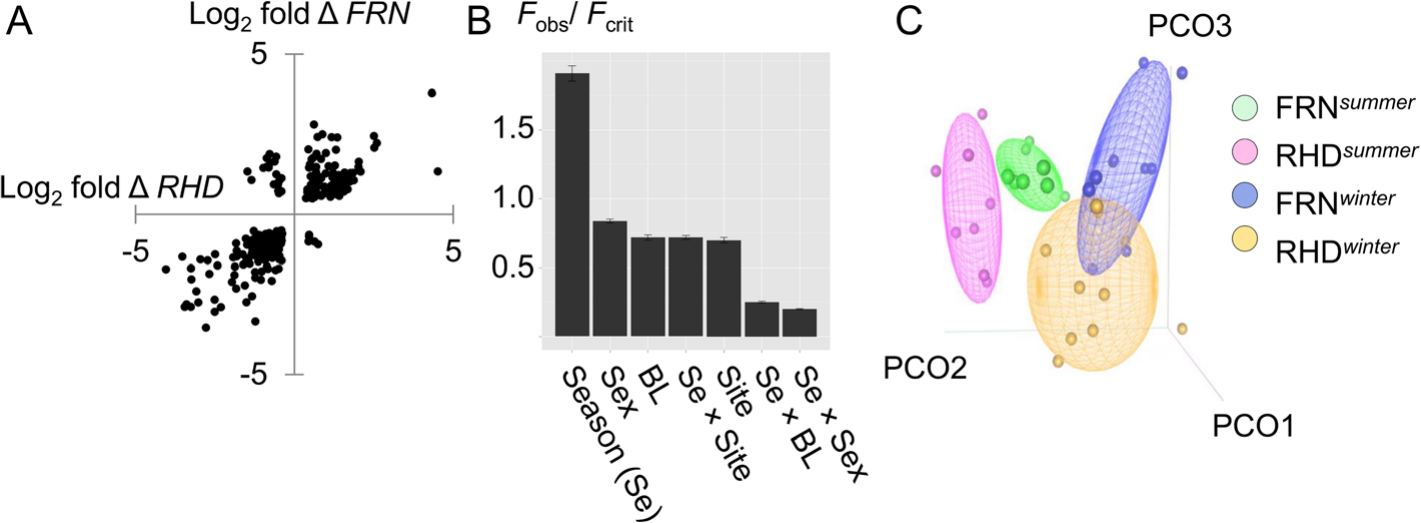
Extremes of season were the dominant predictor of immune gene expression. **a** Scatterplot of log_2_ winter-summer fold expression change (log_2_ fold Δ) for all immune-associated (*ImmPort* list) genes with significant seasonal difference (individual *P* < 0.05) at both RHD and FRN sites. Overwhelmingly such genes were regulated in the same direction across sites. **b** Season was overwhelmingly the most important predictor of immune gene expression, in comparison to site, sex and body size (analysis based on all *ImmPort* list genes, *n* = 3648). Bar chart summarizes results from general linear models (LMs) fitted to each individual log_2_ immune-associated gene expression variable; bars are the mean observed *F* value (± 1 SE) for each model term (BL, body length; Se, season), expressed as a proportion of the critical value (*P* = 0.05) and relate to models lacking interaction terms in the case of the main effects. **c** Principal co-ordinates (PCO) ordination of immune-associated gene expression (all *ImmPort* list genes), indicating strong divergence between summer and winter samples along similar site-specific trajectories; plot showing scatter of individual points against the 3 major axes (PCO-1-3) and concentration ellipsoids containing 50 % of points

Focussing on the immune system we considered genes with predicted orthology to those in the *ImmPort* [17] comprehensive list of immune-associated genes. In total, 244 immune-associated genes out of 3648 were seasonally-biased (differentially expressed in the same direction at both sites). Of these, greater absolute numbers of genes were winter-biased (150) than summer-biased (94). As with the full gene list, immune-associated genes that were consistently seasonally differentially-expressed tended, very strongly, to be synchronously expressed across localities (Fig. 2a).

We also asked how important seasonal influences on the expression of immune-associated genes were in comparison to other sources of variation (site, sex, body size). To answer this we again considered LMs fitted to expression data for all 3648 immune-associated genes, initially with main effects for season, site, sex and body length and then with all 2-way interactions involving season. As for the analyses of genome-wide expression above, the broad pattern in these models was for season to be the dominant influence on immune gene expression (Fig. 2b), compared to individual sex, body size or site. Also, the interactions of season with other terms tended to be small compared to the main effect of season, indicating consistent seasonal effects across site, sex and age. Moreover, PCO ordination of all *ImmPort* list genes (whether seasonally expressed or not) revealed clear differentiation between summer and winter samples along similar trajectories between sites (Fig. 2c). These observations are consistent with overarching temporal environmental drivers acting similarly on the immune system across different habitat types and life-history stages.

### Adaptive immunity genes are summer-biased and innate immunity genes are winter-biased

The 244 consistently seasonally-biased genes from the *ImmPort* list were individually evaluated to identify those with core immunological functions (the *ImmPort* list tending towards inclusivity) (Additional file 7: Table S5). Such “core” genes in the summer-biased set included those involved in, or regulating, adaptive effector response pathways (*rag1, rag2*, *cd8a*, *zap70*, *ccr7*, *il4, igh@ irf4b*, *foxp3b*, *rorc, satb1*), corroborating the summer bias in lymphocyte responses suggested by GSEA analyses. One weakly expressed classical major histocompatibility class (MHC) IIa locus (from chromosome VII [18]) was also detected more strongly in summer, although this was not the case for other more highly expressed MHCIIa loci; the chromosome VII locus is hereafter referred to as *mhcIIa*. There were also summer-biased genes involved in immunological cell adhesion (*itgb2*) and toll-like receptor (TLR)-mediated signalling (*tirap*).

In the winter-biased set there was a lack of genes clearly promoting adaptive immunity. However, there were several genes involved in regulating or suppressing lymphocyte activity (*orai1, apoea, tnfrsf21, bnip3, rnf128, itm2a, tgfbr2*) [19–25]. In addition there were genes associated with innate immune cell activity (*nfkbiz*, *zbtb16b*, *lsp1, cd302*) and interleukin (IL) 1 family signalling pathways (three *il1r* gene cluster members, *il1rap*), and genes like those up-regulated by type I interferons in mammals (*ifi44*/*ifi44l*-like) or involved in TLR signalling pathways leading to the production of type I interferons (*tbk1*). Key elements of non-classical complement pathways (*cfd, masp2*) were also winter-biased.

Although selected on the basis of *Cuffdiff* outputs [26], all of the core immune genes were highly significantly seasonally-biased when analysed in confounder-adjusted LMs with terms for season, site, sex and body length.

### A set of highly co-expressed winter- and summer-biased immune genes can be identified that may lie close to a regulatory axis for seasonal immunity

**Fig. 3:**
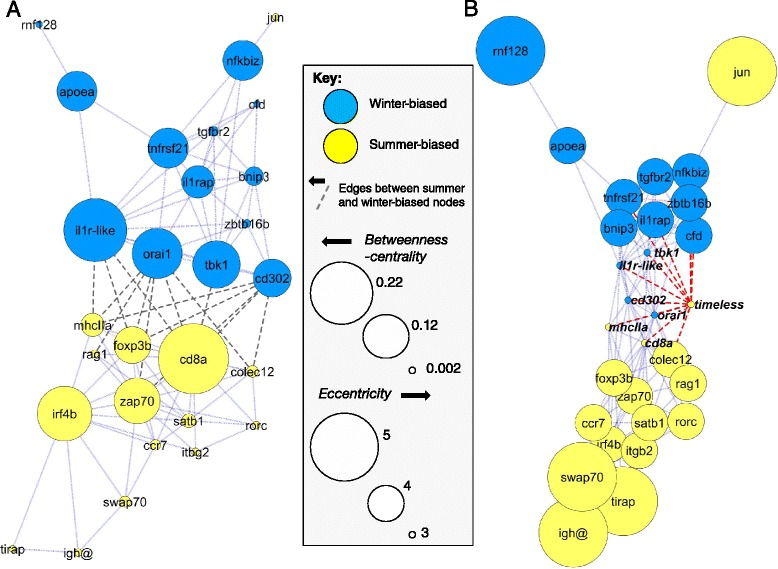
*ARACNe* networks of seasonally-biased core immune genes. Winter- and summer-biased nodes segregate to different regions of networks and interface via a small set of central nodes that are highly connected amongst themselves and also within their respective winter- or summer-biased set. **a** Network 1. Nodes sized according to their betweenness-centrality (a measure of centrality and thus potential influence within a network); network shown with a force-directed layout, modified to highlight edges (dashed) between summer and winter-biased genes (entire network stretched laterally with winter and summer regions displaced from each other vertically). **b** Network 1 re-analyzed with summer-biased *timeless* added as an extra node; nodes sized according to eccentricity (an inverse centrality measure) and shown with an unmodified force-directed layout. Edges from *timeless* connect to winter-summer interface genes and further winter-biased genes

As the regulatory influence of genes on other genes may be reflected in patterns of co-expression, we analyzed these patterns in our transcriptomic data to identify candidate seasonally-biased genes with high regulatory importance. Given the prominence of the seasonal signature characterized above, we used unadjusted data for these analyses in order to preserve the overall context of co-expression. We selected 30 of the seasonally-biased genes with well-defined immune system functions (core immune genes, see above) as hubs for co-expression networks constructed using an information theory based algorithm (*ARACNe*). These hubs included most of the core genes, but excluded those with very strongly linked functions (e.g., *rag1* was included, but *rag2* excluded). In an initial analysis of the hubs alone (Network 1, Fig. 3), summer and winter-biased genes overwhelmingly segregated to different regions of the network, interfacing principally through a relatively small number of genes (“key genes”) that were also highly interconnected within their respective summer or winter-biased sets. This set of highly connected interfacing genes may lie close to the regulatory axis that controls seasonal changes in immune function and comprised *orai1, cd302, tbk1* and *il1r*-like in the winter-biased set and *colec12*, *mhcIIa*, *foxp3b*, *cd8a, zap70*, and *rag1* in the summer-biased set. Remarkably, some of these summer-biased genes (*cd8a*, z*ap70* and perhaps *mhcIIa* [classical chromosome VII locus]) are involved in the T-cell – APC immunological synapse determining T-cell activation. Furthermore, the winter-biased *orai1* codes for a calcium channel that is necessary for T-cell proliferation [27] and that is recruited to the T-cell receptor complex during activation [28]. Loss-of-function mutations affecting mammalian orthologues of six of the genes cause either severe immunodeficiencies (*orai1*, *cd8a*, *zap70, mhcIIa, rag1*) or autoimmunity (*foxp3*), reflecting their potential degree of influence upon the adaptive immune response.

**Fig. 4:**
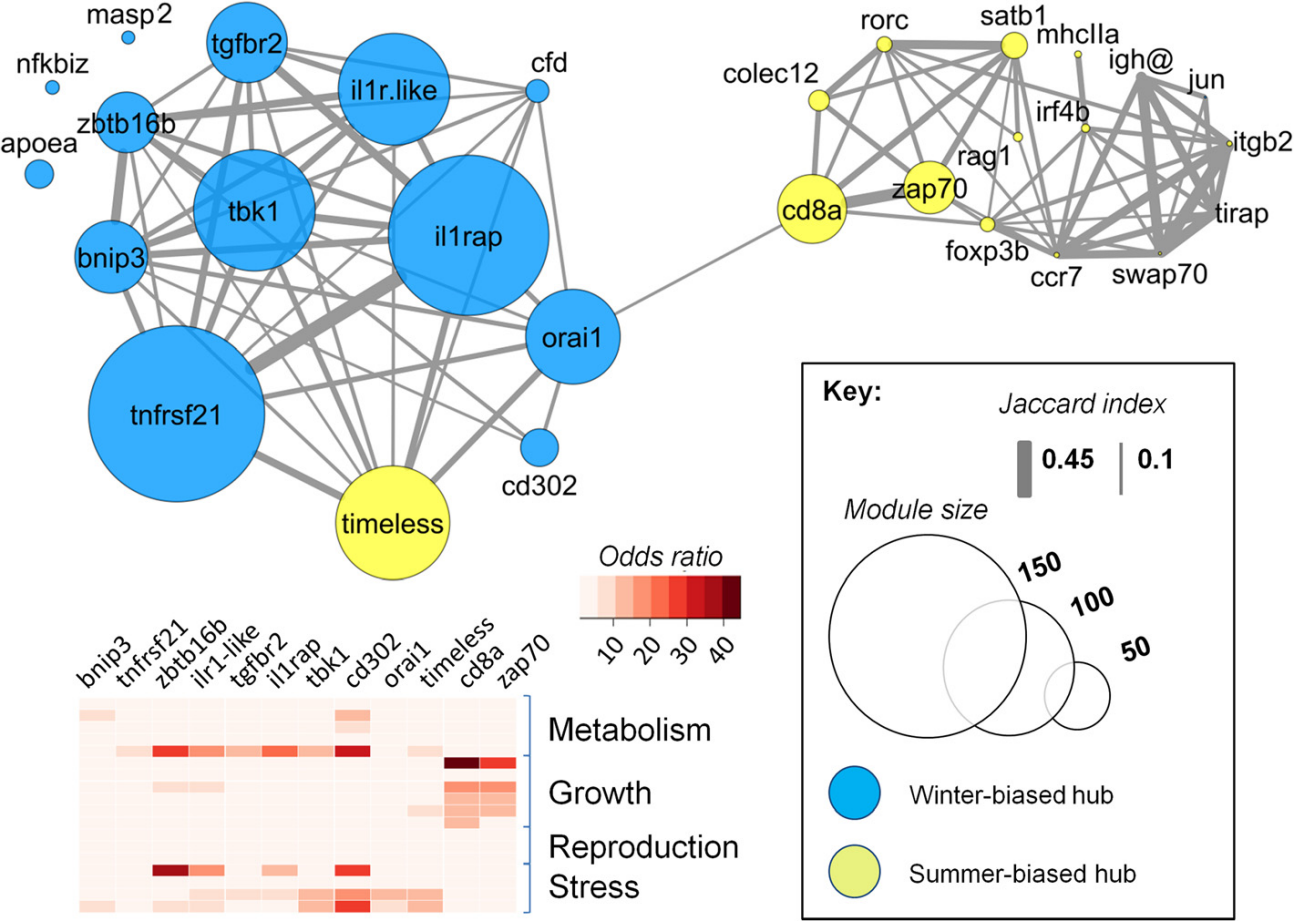
*ARACNe* network (Network 2) of all seasonally-biased genes, specifying core immune genes and *timeless* as hubs. Nodes, shown in a modified force-directed layout, represent gene module sizes associated with hubs and edges are Jaccard similarity coefficients for module composition (cut-off, 0.1). Modules associated with winter and summer-biased immune hubs segregate to different regions of the network, with the strongest winter-summer module similarity between *orai1* and *cd8a*. The module associated with the summer-biased *timeless* is primarily similar to modules associated with winter-biased hubs. For larger modules the heat map (*bottom left*) shows significant odds ratio gene overlaps with gene sets representing organismal signatures of metabolism, growth, reproduction and stress

A different *ARACNe* network (Network 2) including all summer- and winter-biased genes (immune associated and non-immune associated) was also constructed, using the same hub genes as above. When gene modules from this analysis were considered in terms of the degree of gene overlap (Fig. 4), modules associated with winter-biased hubs were largely segregated from those associated with summer-biased hubs. The strongest similarity across winter- and summer-biased modules was between those associated with the hubs *orai1* and *cd8a*, tending to confirm that genes interacting with *orai1* and *cd8a* might be close to the axis of seasonal regulation.

**Fig. 5:**
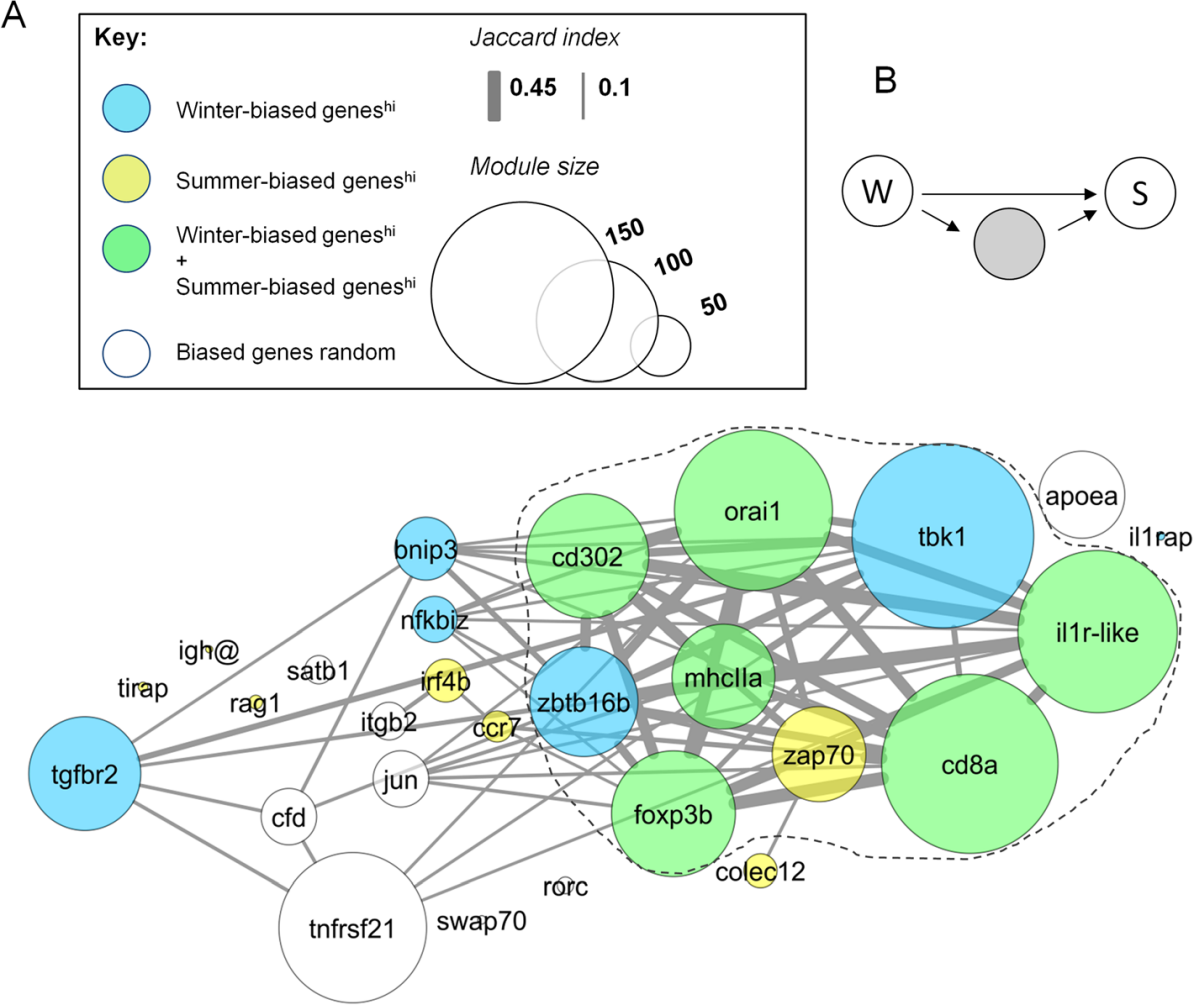
*ARACNe* network (Network 3) of all immune-associated genes (*ImmPort* comprehensive list of immune-related genes), specifying seasonally-biased core immune genes as hubs. **a** Nodes, shown in a modified force-directed network, represent gene module sizes associated with hubs and edges are Jaccard similarity coefficients for module composition (cut-off, 0.1). Node colours indicate modules significantly enriched in winter-biased genes, summer-biased genes or both (see key). Modules associated with hubs that were winter-summer interface nodes in Network 1 tended to be large, to share a high degree of similarity in composition, and to contain significant enrichments of both winter- and summer-biased genes. **b** Form of simple structural equation model (path analysis) used in assessing the influence of winter-summer interface (key) genes from Network 1; W, main axis of covariation in winter-biased core immune genes (represented by the first principal component of a principal components analysis); S, main axis of covariation in summer-biased core immune genes; *grey circle*, expression of individual winter-summer interfacing gene

A third *ARACNe* network (Network 3) was constructed from all immune associated genes (whether seasonally biased or not) and, again, the same set of seasonally-biased immune hub genes used above. In this network, there was a dominant cluster of large, overlapping modules (a meta-module). Most of these were enriched in both summer- and winter-biased genes and associated with hubs that were winter-summer interfacing nodes in Network 1. This dominant meta-module (Fig. 5a) is consistent with the existence of a coherent regulatory unit involved in seasonal immune function.

Finally, we constructed a small (bearing in mind sample size considerations) three-variable structural equation model (SEM) of the form shown in Fig. 5b. We used this to further assess the influence of individual winter-summer interfacing (key) genes from *ARACNe* Network 1 on the seasonal transition in immune function. In this analysis two of the variables were derived as the first components from separate principal components analyses (PCAs) of summer and winter-biased core immune genes (but excluding key interface genes). Each component thus represented the major axis of covariation within the respective summer or winter-biased gene set. The third variable was the expression of a key winter-summer interfacing gene, each of which, in turn, was evaluated in the model. All of the winter-summer interfacing immune genes, except *tbk1*, negated the direct effect of winter-biased on summer biased genes (these were significantly associated in a univariate model) and themselves showed significant associations, of opposite sign, with the summer and winter-biased genes. This supports the linking role of these genes indicated by the *ARACNe* analyses.

### Consistent seasonality confirmed by year-round Q-PCR measurement of key genes over a new annual cycle

**Fig. 6:**
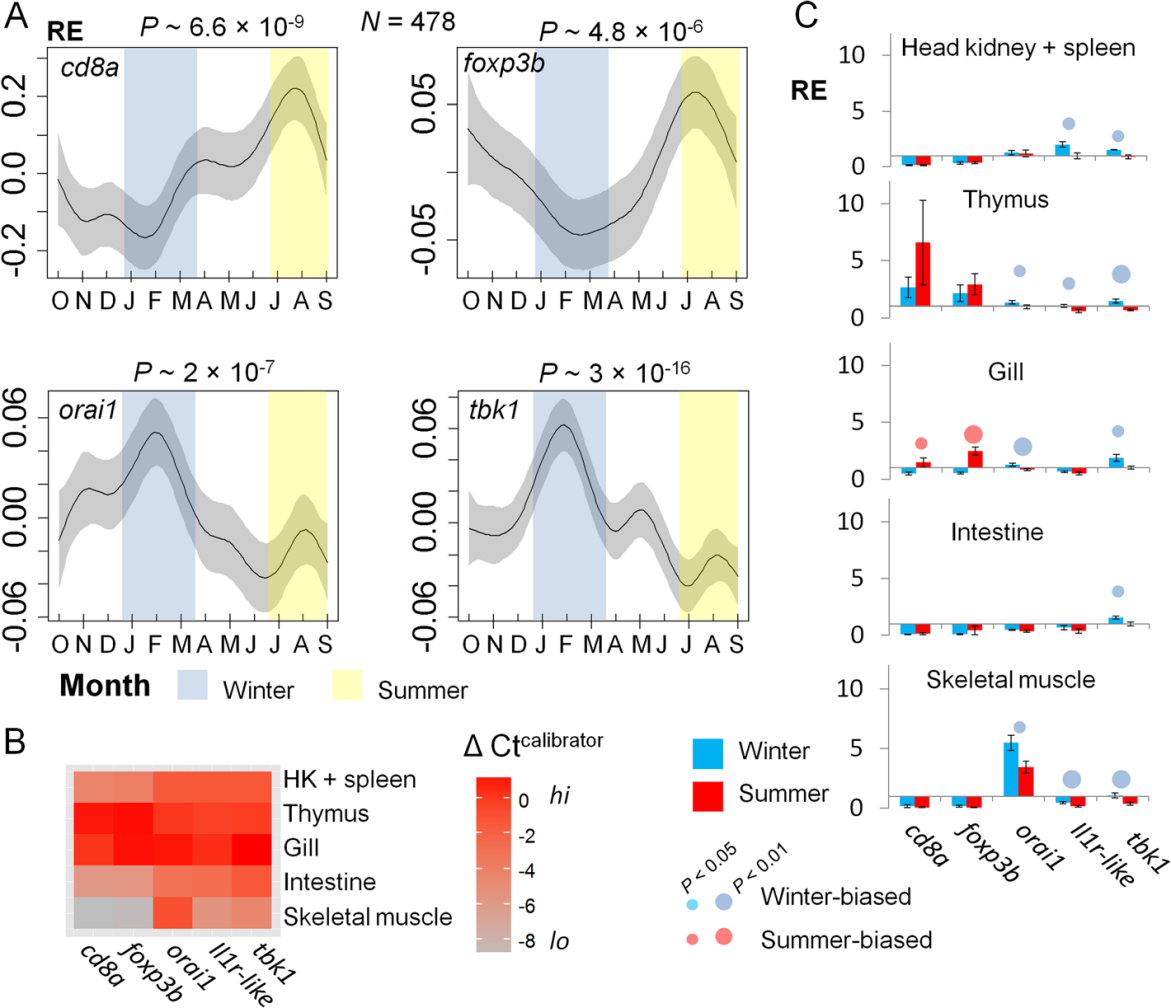
Corroborative whole-fish and tissue-specific Q-PCR gene expression measurements. **a** Temporal variation from October 2013 to September 2014 in whole-fish gene expression for winter-summer interface (key) genes from Network 1, *n* = 478. Relative expression (RE) (normalized to endogenous control genes and indexed to a calibrator sample) is indicated on the y-axis. Plots show thin-plate-spline smoothers for time fitted in a generalized additive model (GAM) with fixed effects for habitat, sex and length; shaded areas represent 95 % confidence regions. Samples were derived from FRN, RHD and artificial outdoors habitats stocked from FRN. The other key gene examined, *il1r*-like, also demonstrated significant seasonal variation (*P* = 0.0019) with peak expression in December (not shown), if log_10_ transformed. **b** Tissue-specific expression of key genes at STO (summer, *n* = 5; winter *n* = 10). Heat map showing relative gene expression across tissues; significant differences occurred for all genes (*P* <0.001). **c** Tissue-specific seasonal variation in key genes at STO. Mean relative expression (RE) ± 1 SE is shown on the y-axis. *P* values (c) relate to directional (1-tailed) *t*-tests of seasonal shifts in the same direction as the whole-fish RNAseq study; 13/25 of these tests were significant but, in comparison (*post-hoc*), 0/25 of 1-tailed tests in the opposite direction were significant. The calibrator sample for tissue-specific analyses was pooled whole-fish RNA from 20 individuals in September. The STO samples showed no significant difference in fish length between winter and summer and were balanced for sex ratio (see Additional file 8: Table S6)

Arbitrarily selecting 5 (*orai1*, *tbk1*, *il1r*-like, *cd8a*, *foxp3b*) from the 10 key genes identified in Network 1 above, we confirmed their seasonal expression bias by quantitative real-time PCR (Q-PCR) in an independent sample set. Whole-fish gene expression measurements were made for 478 individuals sampled in a regular monthly design over a new annual cycle (2013–2014) in our original localities (FRN, RHD) and also in semi-natural artificial habitats populated by stock from FRN. All of the genes showed highly significant confounder-adjusted seasonal patterns (Fig. 6a), overall, with a clear peak in the predicted season.

### Tissue-specific expression of key genes suggests intense seasonality in the gill

We also considered seasonality of key genes (see above) within specific tissues (Fig. 6b, c) at a new locality on the River Stour in eastern England (STO). All of these genes, except for *orai1*, were primarily expressed in organs with known concentrations of lymphoid tissue (Fig. 6b). Furthermore, all of the many instances of significant tissue-specific seasonal bias (13/25 comparisons) occurred in the same direction as predicted by the whole-fish transcriptomic study (Fig. 6c). Outside of the thymus, expression of T-cell-associated genes (*cd8a*, *foxp3b*) was highest in the gill, lower in head kidney, spleen and intestine and negligible in skeletal muscle (Fig. 6b, c). This is consistent with a strong concentration of T-cell activity in the gill. Moreover, the summer bias of T-cell-associated genes was seen primarily in the gill (Fig. 6c). In the case of *orai1*, whose expression is important in mammalian T-cells [27] but not narrowly characteristic of them, high expression occurred in skeletal muscle (consistent with a known physiological importance in this tissue [29]) (Fig. 6b, c). This gene was, however, also robustly expressed (Fig. 6b) and winter-biased (Fig. 6c) in the organs with greatest T-cell-specific expression, thymus and gill, supporting a possible role in seasonal immunoregulation. Genes from innate signalling pathways (*tbk1*, *il1r*-like) tended to be winter-biased in all tissues (Fig. 6c). Overall, gill most closely reflected the pattern of seasonal bias seen in whole-fish mRNA pools (Fig. 6c).

### Seasonal immune gene expression links to wider life history signatures

Some seasonal immune functions in vertebrates are controlled by photoperiodic time measurement [30, 31] and the circadian molecular clock may also have a role in co-ordinating circannual biological rhythms [2, 32]. A scan of the seasonally-biased genes for those involved in such processes revealed that *timeless* (a clock-associated gene) occurred within the summer-biased set. When added to the *ARACNe* networks above *timeless* was, remarkably, most strongly connected to key winter-summer interface genes, with more connections to winter-biased genes (Fig. 3b). However, other genes involved in clock machinery or photoperiodism did not show the same tendency and *timeless* is known to have physiological functions in mammals that are independent of any role in biological clocks [33].

**Fig. 7:**
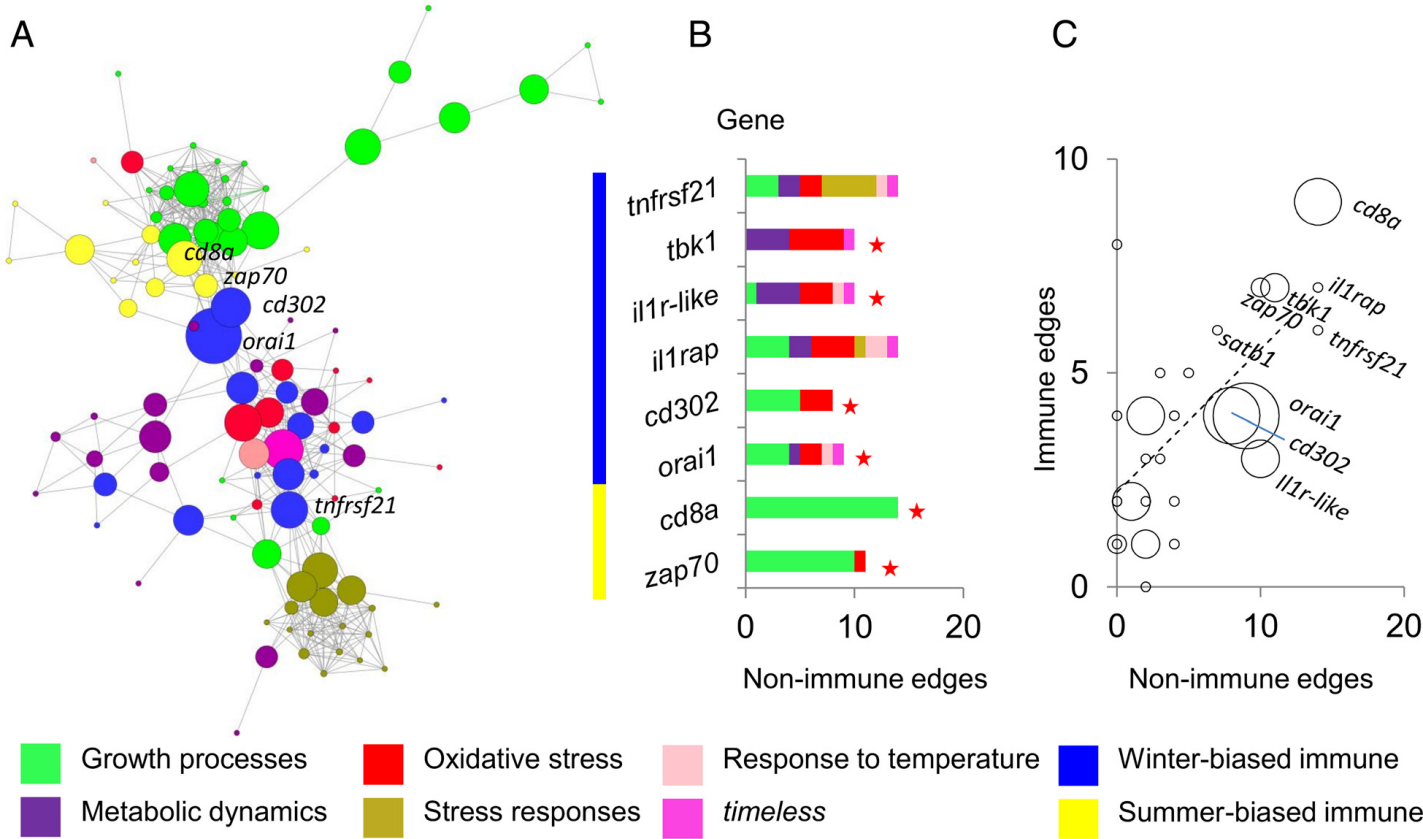
Association between seasonal core immune genes and wider organismal signatures of growth, metabolism and stress. **a** *ARACNe* network (Network 4) including seasonal core immune genes, *timeless*, and seasonal genes from curated sets representing growth, metabolism, and aspects of stress (oxidative stress, stress responses, temperature responses); specifying all genes as hubs; nodes sized according to their betweenness-centrality. Network shown in an unmodified force-directed layout. **b** Bar chart showing, for the most highly connected core immune genes in Network 4, the distribution of edges with genes representing wider organismal signatures (stars indicate winter-summer interface “key” genes in Network 1). Colour bar outside vertical axis indicates winter- or summer expression bias. **c** Scatterplot showing, for core immune genes in Network 4, numbers of edges to other immune genes *vs* numbers of edges to non-immune genes (Pearson *r* = 0.65, *P* = 1.6 × 10^−4^); point sizes are proportional to the number of winter-summer edges for the gene in Network 1 (key genes from Network 1 were significantly more likely to show > 7 non-immune edges in Network 4, compared to other core immune genes, *P* = 0.006)

In order to place the seasonal variation in immune pathways within an even wider organismal context, we next tested the larger gene modules from the *ARACNe* network of all seasonally-biased genes (Network 2) for overlap with the gene sets representing other life history components and responses to stressors (Fig. 4; see Additional file 3: Table S3). The modules formed by summer-biased hubs *cd8a* and *zap70* contained genes associated with organismal growth. In contrast, modules for winter-biased hubs contained genes associated with metabolism and responses to stress (oxidative stress, organismal stress and temperature change). To explore these associations further, we then constructed an additional network (Network 4) including seasonally-biased core immune genes, *timeless*, and representatives from the wider organismal gene sets. The latter were selected on the basis that they were relatively differentially seasonally expressed in GSEA analyses. In the resulting network (Fig. 7), in which all genes were specified as hubs, those representing growth processes were again associated primarily with summer-biased adaptive immune genes (especially *cd8a* and *zap70*). Apart from some connections to growth-related genes (especially for *orai1* and *cd302*), mostly the edges emanating from winter-biased genes were to *timeless* and to genes representing metabolism and oxidative stress. Notably, winter-summer interfacing (key) genes from Network 1 showed especially high numbers of non-immune edges (statistical associations) in Network 4 (Fig. 7a, c) (and there was a general tendency for immune genes with many edges to other immune genes to also have many edges with non-immune genes). As connectivity may reflect regulatory influence, this reinforces the potential regulatory importance of key winter-summer interface genes from Network 1. In fact, only two non-interface genes showed high numbers of non-immune edges in the network. These were the innate winter-biased genes *il1rap*, which showed a pattern of edges similar to *il1r*-like, and, more distinctively, *tnfrsf21*, which was the main immune node with connections to general stress responses.

### TLR signalling pathways show seasonal modulation

**Fig. 8:**
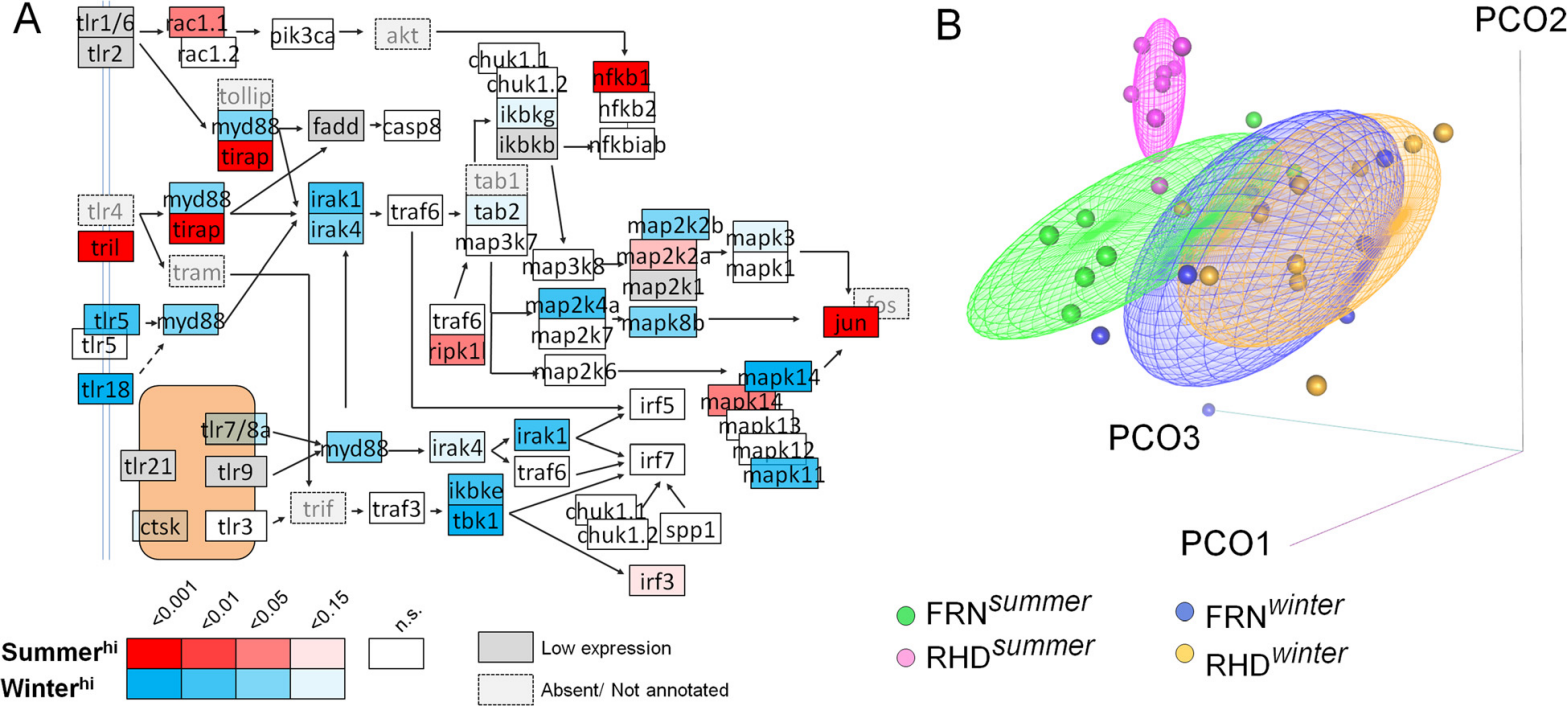
Seasonal bias in toll-like receptor (TLR) signalling pathway. **a** Differential winter-summer gene expression in pathway members (based on modified KEGG TLR signalling pathway), demonstrating winter bias in some cases and summer bias in others. Categorization of differential expression is based on overall significance levels in general linear models (LMs) with explanatory terms for site, sex and length (less stringent criteria than used in initial genome-wide analyses). **b** Principal co-ordinates (PCO) ordination of gene expression in all pathway members, revealing considerable winter-summer discrimination; scatter of individual points against the 3 major axes (PCO-1-3) and concentration ellipsoids containing 50 % of points

Finally, given the importance of *tbk1* in gene co-expression networks (and its role in TLR-mediated signalling) and also the contrasting seasonal expression of *tirap* (associated with other TLR-mediated signalling pathways) we examined TLR signalling pathways in more detail (Fig. 8a). This used genes from the KEGG TLR-signalling pathway supplemented by a few further *a priori* selected genes associated with TLR function in fishes (*tlr18*, *tlr21*, *tril*). There was a clear seasonal modulation. Most seasonally-biased genes (including *myd88* and toll-like receptors 5, 7, 8 and 18) tended to be winter-biased, although some (e.g., *tril*, *tirap*, *nfkb1*) were strongly summer-biased. There was considerable discrimination between summer and winter samples when TLR signalling gene expression was ordinated using PCO (Fig. 8b).

## Discussion

We have demonstrated seasonal re-adjustments of immune system gene expression in naturally-occurring freshwater teleosts. These occurred most intensely in the gill and were substantial (greater than variation between habitats and life-history stages) and over-arching (with consistent trajectories across habitats and life-history stages). In keeping with some previous suggestions about seasonal immune function in teleosts [34], we found that genes marking adaptive immune processes were summer-biased (expressed more strongly in summer), whilst certain innate immune genes were winter-biased. However, as set out below, our observations provide considerable new insights into the control of seasonal immune responses.

Transcriptomic analyses (based on whole-fish samples) indicated that summer-biased genes included many centrally involved in lymphocyte responses. For example, the recombination activating genes (*rag1*, *rag2*) and genes associated with particular adaptive cell populations: T-cells (*zap70*), cytotoxic T-cells (*cd8a*), helper T-cells (*foxp3b*, *il4*) and B-cells (*igh@*). In contrast, the set of winter-biased genes lacked those promoting adaptive effector responses. In all cases, winter-biased genes associated with T- or B-cell responses were regulatory or even suppressive in nature. This strongly suggests a regulatory control of adaptive immunity during winter, rather than, or additional to, a loss of function due to the kinetic consequences of low temperature in a cold-blooded organism. Furthermore, there were gene expression signatures of elevated innate immune functions in winter: including IL-1 signalling and non-classical complement pathways. A complex modulation of genes involved in innate TLR-mediated signalling occurred, with a predominant winter bias.

We designed the sampling for our transcriptomic study to, as far as possible, reduce correlation between season and ontogeny, and we also carefully considered, *post-hoc*, the possible role of ontogeny in generating apparent seasonal differences. To ensure that an extensively overlapping range of effective ages was present in our winter and summer transcriptomic samples, we deliberately selected a wide range of fish sizes within samples (to the extent that there were no significant differences in length between winter and summer samples). Through monitoring the growth of fishes in artificial outdoor habitats we confirmed that age predicted the majority of variation in length, and we adjusted for length, as a surrogate for age, in statistical models applied to transcriptomic data. Importantly, the much greater overall signature of season compared to length in statistical models applied to genome-wide and immune system-wide gene expression is not consistent with ontogeny being a major confounder in our study. Moreover, the balancing of size across seasonal samples, and the adjustment for length in our statistical modelling, also accounts for the possibility that growth allometries in different tissues (for example, proportionately increased muscle mass with size) may have biased results for whole-fish samples.

We also considered whether the patterns of gene co-expression in our transcriptomic data could give insights into the regulation of seasonal immune function. Information theory-based network analyses [35] of expression in seasonally-biased core immune system genes identified a small set of genes lying at the interface between summer- and winter-biased genes. These were highly networked (statistically associated) amongst themselves and also each highly networked within the seasonally-biased group to which they respectively belonged. Remarkably, several of the interfacing genes have roles in APC-T-cell immunological synapses (*cd8a*, *zap70*, *orai1* and perhaps the summer-biased classical *mhcIIa* locus [27, 36–38]) and mutations leading to loss of function in their mammalian orthologues cause primary immunodeficiencies [27, 36–38]. Also amongst these interface genes, *foxp3* [39] is a master regulator of regulatory T-cell function and recombination-activating genes are central to the production of re-combined adaptive receptors [40]. In mammals loss-of-function mutations in these genes respectively cause lethal autoimmunity and severe combined immunodeficiency [39, 40]. Other interfacing genes are involved in innate processes that might precede antigen presentation: innate signalling pathways (*tbk1*, *il1r*-like [41–44]) and antigen internalization via phagocytosis or endocytosis (*cd302, colec12* [45, 46]).

When we added the summer-biased clock-associated gene *timeless* to these networks, it proved to be closely associated with interface genes, and especially with winter-biased (mostly innate) interface genes. Whilst this could reflect some co-ordination via a seasonal oscillator, though, other genes involved in photoperiodism or circadian rhythms did not enter the networks in corresponding ways. It is also the case that *timeless* itself has a relatively poorly resolved role in the mammalian circadian clock and is known to have independent physiological functions [33] (consistent with the links to metabolic pathways discussed next).

During winter there was a genome-wide signature indicative of elevated metabolic processes and metabolite transfer and organismal stress, and in summer a signature of growth and developmental processes. Again using network analyses of our transcriptomic data, we finally asked how seasonal changes in immunity might be related to this background. We found that the winter-summer interfacing (key) immune genes identified above were especially highly connected to genes involved in non-immune seasonal variation, further emphasizing their relevance in the seasonal control of immunity. Genes involved in metabolism and oxidative stress interconnected densely with winter-biased innate genes, and amongst these especially to the winter-summer interfacing genes *tbk1* and *il1r*-like, and also to *il1rap*. On the other hand, genes involved in general organismal stress responses linked differently to winter-biased immune responses: primarily via *tnfrsf21*, a protein that triggers apoptotic pathways [47] and restrains T-cell [48] and B-cell [49] responses. In comparison, genes involved in non-immune summer signatures (growth and development) networked primarily to summer-biased adaptive genes, especially to the summer-biased interface genes *cd8a* and *zap70*. These observations suggest an unexpectedly strong link between growth processes and adaptive immunity and that one, or both, may favour permissive conditions for the other. Taken together, the above patterns indicate that multiple organismal processes are likely to interact with the seasonal regulation of immunity, additional to the possible influence of any “hard-wired” circannual oscillator. It might be expected, then, that predictable seasonal influences will be modified by less predictable non-cyclical temporal variations in environmental stressors [30].

To validate our transcriptomic analyses we returned to our original study localities, an upland lake and river in mid-Wales, and also considered artificial outdoors habitats stocked from the lake site. Using Q-PCR measurements we confirmed (with very strong statistical support) seasonality in a panel of the key immunity genes predicted to be winter- or summer-biased. This year-round monthly analysis considered whole-fish samples (*n* = 478) and was carried out in a new annual cycle that lacked unusually cold winter or spring weather. Furthermore tissue-specific analyses (discussed below) at an entirely new locality (a euryhaline estuarine site in eastern England) found that all tissues showed seasonal expression changes and these changes all occurred in the same direction as in the whole-fish studies at our original sites. Thus, overall we considered 3 very divergent localities (upland lake, river, estuarine) across 2 years and found compelling evidence to support a general pattern such as that indicated in our initial transcriptomic measurements.

In addition to our analyses of whole-fish mRNA pools, we also confirmed tissue-specific expression patterns through Q-PCR measurements of key immunity genes at a new estuarine locality (considering head kidney, spleen, thymus, gill, intestine and muscle). As indicated above we found many significant tissue-specific seasonal expression differences and all of these were in the direction predicted by our other whole-fish studies. The most pronounced seasonal expression profile occurred in the gill (and this profile most closely reflected seasonal change at the whole-fish level). Furthermore, the gill contained the most intense concentration of T-cell activity outside of the thymus, with elevated expression of T-cell associated genes such as *cd8a* and *foxp3b*, and expression of these genes was seasonal in the gill but non-seasonal in the thymus. These observations are consistent with the known responsiveness of immune gene expression in the teleost gill to environmental stimuli [50], and also with the recent discovery and characterization of extensive, T-cell rich, interbranchial lymphoid tissue (ILT) in teleost fishes [12, 51–53]. Our results suggest the possibility that ILT may have an important role in seasonal immune function.

Finally, and whilst the present study is intended to characterize the seasonal dynamics of gene expression, rather than identify environmental causation, we briefly consider what external agents may drive the responses that we observed. In highly seasonal temperate zone habitats, such as the ones we consider here, each of temperature, diet, photoperiodic responses, pathogen exposures, or other biotic or abiotic manifestations of the environment, could be involved to unknown degrees. In the future, by matching detailed field observations with mesocosm studies and laboratory experiments, we expect to dissect the relative contributions of these influences to seasonal immune variation and to the immune phenotype more generally.

## Conclusions

Our results suggest that in wild teleosts, during winter conditions, adaptive immune activity declines in a manner that involves the expression of regulatory genes affecting lymphocyte function. This is indicative of a controlled, strategic response rather than a simple kinetic tracking of environmental temperature. Seasonal change is most prominent in the gill, suggesting ILT may be important in such responses. Further broad attention to seasonal immune function is certainly warranted, given the likely practical relevance – through effects on infectious disease susceptibility and inflammatory status – to health in humans and domesticated animals and to fitness in natural populations.

## Methods

### Sampling and habitats

Samples of three-spined sticklebacks (*Gasterosteus aculeatus* L.) for transcriptomic analysis were taken at 9:00–12:00 h (UTC) in September 2012 and in March 2013, outside of the breeding season and respectively prior to the autumnal and vernal equinoxes. Specimens were collected at two contrasting sites in the Ceredigion area, mid Wales, U.K. (8–10 individuals/site/sampling point). One site (FRN) was a 7.2 ha upland lake, Lake (Llyn) Frongoch, 13.7 km from the sea at an elevation of 280 m (52.3599,–3.8776). The other (RHD) was a non-tidal minor channel of the River (Afon) Rheidol, 3.5 km from the sea at an elevation of 10 m (52.4052,–4.0372).

Additional specimens for corroborative tissue-specific quantitative real-time PCR (Q-PCR) gene expression studies (September 2012, *n* = 5; March 2013, *n* = 10) were collected from a site (STO) on the river Stour in Sussex, U.K. (51.9544, 1.0222). The STO site was in a small, tidal side-channel of the main river at an elevation of 1 m and 2.2 km inland from the tidal sluice opening into the main estuary.

U.K. meteorological office records indicate that March 2013 encompassed extended winter conditions and was the coldest U.K. March since 1962 and joint second coldest since 1910 [54]. Weather patterns in September 2012 were unremarkable for the time of year. Water temperatures at the study sites varied across an approximate range of 13–20 °C in September and 0–5 °C in March; the FRN and STO samples in March were collected from habitats with superficial ice formation.

To avoid the confounding of variation between September and March with individual ontogeny, all samples were selected to contain a wide (extensively overlapping) range of sizes. Sample characteristics are summarised in Additional file 8: Table S6 (and there was no significant winter-summer difference in length for any of the locality-specific sample sets). Given considerations of timing and environmental temperature, the (widely overlapping) potential effective age variation in our samples is set out in Additional file 4. The use of body size as an age indicator is validated by data from our outdoors artificial habitats (see below), where time explains a minimum of 57 % of variation in individual body length over a 12 month study interval, even given a heterogenous starting population of wild fishes that varied in length by a factor of up to × 1.6 (see Additional file 4: Figure S1). Thus, it was possible to partition the effects of age in statistical models (described below) through the inclusion of body length as a surrogate term.

In addition, and also for the purpose of corroborative Q-PCR gene expression measurements, we considered samples of fishes from FRN (~10 individuals/month), RHD (~10 individuals/month) and 12 outdoors artificial 300 L habitats (~20 individuals/month) from October 2013 to September 2014. The artificial habitats were located on the Aberystwyth university campus and stocked in August-September 2013 with post-larval fishes from FRN which were given 2 × anti-parasitic Praziquantel treatments (24 h at 4 mg l^−1^; FlukeSolve, Fish Treatment Limited) to prevent *Gyrodactlylus* epizootics and maintained on a diet of frozen mini bloodworm (Tropical marine centre) adequate for normal growth. Water temperature within the artificial habitat units was uncontrolled, or was controlled a small increment (2 °C) above the ambient temperature in adjoining uncontrolled units. U.K. meteorological office climate summaries [55] confirm that this 2013–2014 sampling period occurred across average autumn temperatures, above average winter and spring temperatures, lacking frost conditions, and a summer period lacking extremely hot weather.

All animal maintenance and sampling of animals in the field followed U.K. Home Office (HO) regulations and local (Aberystwyth University) ethical procedures.

Whilst parasite infections are not considered explicitly in the present study, the river (RHD) and lake (FRN) populations studied supported divergent and predictable macroparasite communities (with limited seasonality) (unpublished data), whose differential influences are likely to emerge primarily in the site effect of analyses described below. There was no evidence of infection or pathology in any of the specific organs used for tissue comparisons.

### Sample handling, nucleic acids processing and library preparation

Sticklebacks were captured individually using a dip net and immediately killed by concussion and de-cerebration and stored in *RNAlater*™ at ambient temperature. On return from the field (within 1–2 h) the samples were transferred to 4 °C overnight and then to -80 °C for long-term storage. Immediately prior to RNA extraction, sticklebacks were thawed at 4 °C, dabbed dry with tissue and weight (mg) and standard length (mm) recorded. For transcriptomic studies, RNA from whole fishes was extracted using the *Isolate II RNA mini kit* (Bioline): whole individual fishes were homogenized in lysis buffer using a 5 mm stainless steel bead (Qiagen, 69989) in a Qiagen TissueLyser LT system and a standard aliquot of the homogenate passed through the manufacturer-recommended protocol. RNA extracts were subjected to standard quality control diagnostics and individually barcoded cDNA libraries (mRNA focussed) for 36 fishes were prepared using the *TruSeq RNA Sample Preparation Kit v2* (Illumina).

For Q-PCR, RNA was extracted from the 2013–2014 monthly samples as above, whilst RNA from the STO samples was extracted using the *RNAqueous-Micro Total RNA Isolation Kit* (Life technologies). All samples were DNase treated prior to conversion to cDNA with the *High Capacity RNA-to-cDNA Kit* (Life technologies).

### Next generation sequencing and differential expression analysis

Individually barcoded *Truseq* sequencing libraries were sequenced using 4 lanes of an Illumina HiSeq2500 sequencer at IBERS, Aberystwyth University. Libraries for 2 summer individuals and 2–3 winter individuals from each locality were run on each lane (thus balancing different sampling units across lanes). Following removal of adaptors, the output paired end reads (~110 bp) were quality-controlled using *FastQC* [56] and the leading 10 bp of all reads trimmed prior to analysis via the *Cufflinks* suite of programmes [26]. Reads were mapped to the stickleback genome (Broad, gasAcu1) using *Tophat* and *de novo* assembled into transcripts using *Cufflinks*. Transcripts from all samples were merged with *Cuffmerge*, using the USCS genes annotation (for gasAcu1) as the reference annotation. Differential gene expression analyses were run for each of the sites separately using *Cuffdiff* with parameters set for geometric library normalization, pooled dispersion estimation, a false discovery rate of 0.05, a minimum alignment count of 10, and using multi-read correction and bias correction. For subsequent analyses, FPKM data for individual loci were generated with *Cuffnorm*. Predicted genes with <0.5 FPKM mean expression or >50 % undetectable expression were excluded from all analyses below.

### Q-PCR gene expression measurements

Quantitative real-time PCR (Q-PCR) gene expression measurements were carried out in a 2-step format with SYBR Green chemistry. Primers sets (Table 1) used to assay target (*il1r*-like, *tbk1*, *orai1*, *cd8a*, *foxp3b*) and endogenous control genes all featured intron-spanning primers and were determined to be 100 ± 10 % efficient under reaction conditions. Samples were run in a 384-well plate format on a Life technologies QuantStudio 12 K flex real-time PCR system (2013–2014 monthly samples) or in a 96-well plate format on a Life technologies StepOnePlus real-time PCR system (STO samples). All assay plates included a calibrator sample run in triplicate, unknown samples run in duplicate and no-template control wells. A proportion of samples were also processed with reverse-transcriptase negative controls. For the 384-well plate assays, each assay plate was pipetted by an Eppendorf epMotion M5073 robot. Endogenous control genes, *yipf4* and *acvr1l*, were selected as an optimally stable pairing for whole-fish analyses by the *NormFinder* algorithm [57] applied to the RNAseq expression data (FPKM). Genes entered into this analysis were previously filtered from the genome-wide set by a lack of seasonal expression bias at both RHD and FRN, an overall coefficient of variation <12 %, detectable expression in all samples, and mean FPKM > 5. Genes *yipf4* and *acvr1l* were also used as endogenous controls for within-tissue expression comparisons in different tissues (spleen and head kidney, thymus, gill, intestine, skeletal muscle). A lack of tissue-specific seasonal variation in these genes was indicated by the fact that, for each tissue, threshold cycle (Ct) difference with respect to the same gene in the calibrator sample (Ct gene *x*_i_ calibrator – Ct gene *x*_i_ sample), ΔCt^calibrator^, did not show significant winter *vs* summer variation in general linear models (LMs) with explanatory terms for season and 3 measures of sample quality (log_10_ RNA concentration [ng μl^−1^], 260 nm/280 nm absorbance ratio, 260 nm/230 nm absorbance ratio and their quadratic terms). For assays normalized to endogenous control genes (2013–2014 monthly samples and within-tissue comparisons in STO samples), relative expression (indexed to the calibrator sample) was calculated using the ΔΔCt method. For expression comparisons between tissues (STO samples), normalization to endogenous control genes was not used as invariant expression of individual genes across many tissues is unrealistic. In these cases tissue-specific expression was derived as the ΔCt^calibrator^ values predicted for each tissue by a linear mixed model (LMM) with a random term for individual fish and fixed terms for tissue, season and sample quality measures (as above). An alternative approach to normalization in the between tissue comparisons, using global normalization (normalization of each gene to all of the other genes), produced a similar interpretation. Calibrator samples were created by pooling cDNA aliquots from all individual samples for the 2013–2014 monthly analysis, and by pooling equal cDNA aliquots from 20 whole-fish (sampled in September) for the STO analysis.

**Table 1:** Primers used for quantitative real-time PCR (Q-PCR) measurements

Gene	Ensembl gene number	Primers
*cd8a*	ENSGACG00000008945	F - CCACCCTGTACTGCAATCGA
		R - CCGCCTGCTGTTTTCTTTTG
*foxp3b*	ENSGACG00000012777	F - TCTGAACACAGTCATGGGGAGA
		R - CCAGGATGAGCTGACTTTCCA
*orai1*	ENSGACG00000011865	F - GCACCTCGGCTCTGTTGTC
		R - CCATGAGGGCGAAGAGGTGTA
*tbk1*	ENSGACG00000000607	F - AGACGGAGCAGCTGTTCGA
		R - GCATATCTCATCATATCTGACGACAT
*il1r*-like	ENSGACG00000001328	F - GAACGCGAGAACTGCAAGAAC
		R - GGGACGCTGGTGAAGTTGAA
*acvr1l*	ENSGACG00000010017	F - CACTTTAGCGGAGCTGTTGGA
		R - AGAAAAGGAAGTCCGGAACCA
*yipf4*	ENSGACG00000002189	F - CCCTCAAACGGAGACTTTACGT
		R - GGTGCCGCTGAGCTCTTC

### Analyses of RNAseq data

As a small preliminary exercise to assess the relevant information content of the Broad gasAcu1 assembly for our wider study, we arbitrarily selected a panel of 31 immunological genes-of-interest whose existence would be expected to be conserved in a lower vertebrate genome and searched for these in the Ensembl stickleback database and the gasAcu1 genome assembly. From this list, 26 (84 %) were associated with predicted database genes (annotated), 3 (10 %) were detectable in the genome assembly but not annotated in the database and 2 (6 %) were absent from the database and genome assembly. In all cases fragments of the genes (confirmed by sequencing) were amplifiable by PCR using primers designed from the assembly sequence, or, in the case of the two missing genes, fragments (also confirmed by sequencing) were amplified using primers designed from conserved regions in multi-species (teleost) alignments. After filtering out low expression genes (see above), the RNAseq dataset contained 20947 predicted loci. Of these, 16575 (79 %) matched genes in the Ensembl *G. aculeatus* database. These data suggested that although a proportion of real genes were missing in the stickleback genome assembly, sufficient information was present to take a broad view of genome-wide expression patterns.

Genes were classified as summer- or winter-biased if they were significantly biased in the same direction at both RHD and FRN at an individual error rate (*P* < 0.05) based on *Cuffdiff* output; this represents a combined individual *P* <0.0025, in practice a more stringent cut-off than a False discovery rate (FDR)-adjusted *P* = 0.05 threshold for a single locality. For unannotated predicted loci that were seasonally-biased we performed a series of standardized *Blast* (*tblastx*) searches that identified a small number of additional genes with high confidence.

For analyses involving comparisons to curated gene sets, we used Ensembl Biomart [58] to convert the identifiers for annotated stickleback genes to the HGNC symbol for an estimated orthologous human gene. Where there were multiple predicted human orthologues (typically related in broad function), a single estimated orthologue was randomly retained per stickleback gene. Similarly, where more than one stickleback gene shared the same predicted human orthologue, only one of these was randomly retained in the gene list. Thus only annotated stickleback genes with a corresponding predicted human orthologue and HGNC symbol were considered in these analyses.

Immune-associated genes were initially defined by homologous relationships with genes from the relatively inclusive *ImmPort comprehensive list of immune-related genes* [17]. Seasonally-biased genes from this list were individually, manually assessed for core immune functions (evidence of direct involvement in immune effector or regulatory activity) using links from the gene list report in *DAVID* 6.7 and from *GeneCards* v. 4.0.

Gene set nrichment analysis (*GSEA v2.1.0*) [59, 60] was used to investigate whether *a priori* defined gene sets showed significant expression differences between winter and summer samples. Separate GSEA analyses were carried out for FRN and RHD (Fig. 1a), using ranked winter-summer expression changes (*GSEAPreranked*), and comparing to all KEGG (c2.cp.Kegg.v.5.0.symbols.gmt) and REACTOME (c2.cp.Reactome.v.5.0.symbols.gmt) pathway gene sets within the *MsigDB* database [60]. FDR-adjusted *P* values from this analysis were combined for the two localities by Fisher’s method [61] and combined FDR *P* values < 0.05 were considered further. In parallel, more targeted GSEA analyses were carried out using smaller numbers of selected REACTOME and GO gene sets to represent different immunological processes and also wider organismal processes (growth, responses to stress, metabolism, reproduction). Applying hypergeometric distribution tests (Fisher’s exact test) for overlap between gene sets, these selected gene sets were additionally used to probe the sets of winter-biased and summer-biased genes identified above, and sets of genes from modules identified by network analyses (see below).

The information-theory (mutual information, MI) based programme *ARACNe2* (Algorithm for the construction of accurate cellular networks) [62] was applied to predict networks of interactions between gene products. Simulation studies [63] indicate this approach retains useful accuracy at sample sizes of the order utilized here (*n* = 36 fishes with transcriptomic data). Working with log_2_-transformed data we constructed the following networks: for seasonally-biased core immune genes alone (Network 1); for the full genome-wide set of seasonally-biased genes (Network 2); for the full set of *ImmPort* list immune-associated genes, whether these were seasonally-biased or not (Network 3); for the core-seasonally-biased immune genes and, in addition, groups of genes from the sets we used to represent wider organismal processes (selecting those that tended to be seasonally-biased in GSEA analyses) (Network 4). In Network 1 and 4 all genes were set as hubs, whilst in Networks 2 and 3 the seasonally-biased core immune genes were set as hubs. Networks were constructed with the adaptive partitioning algorithm, using a mutual information (MI) threshold estimated by a pre-processing run. For the networks shown, *P* thresholds were set at 1 × 10^−5^ (Networks 1 and 3), 1 × 10^−4^ (Network 2) or 1 × 10^−6^ (Network 4), with correction for the number of markers in the case of Networks 1 and 4. All networks shown were bootstrapped (2000 resamples; significance cut-off for reported edges, *P* = 1.0 × 10^−6^). *Cytoscape* 2.8 was employed to visualize networks (initially using force-directed layouts, from which the layout of nodes was sometime modified for clarity of presentation) and to calculate network statistics (*Network Analyzer* plugin). Betweenness centrality [64] was calculated to represent the centrality of nodes within a network (and thus the tendency of indirect connections across the network to route via that node). Eccentricity, the maximum path length connecting a node to any other node in the network, was also calculated to (inversely) reflect nodes that lie at the centre of a network. Both of these quantities might be indicative of the regulatory influence of individual nodes by reflecting their tendency to be co-expressed (and thus perhaps co-regulated) with many other nodes [65].

Principal co-ordinates analysis (PCO) of log_2_ transformed FPKM gene expression values was employed to ordinate individuals across other study variables (*LabDSV* package, *R*). First principal component scores from principal components analyses (PCAs) on the correlation matrix were used to represent the major axis of covariation within genes sets in some analyses. The *R* package *GeneOverlap* was used to compute overlap statistics amongst gene sets (significance tests based on a hypergeometric distribution, odds ratios and Jaccard similarity indices). For general linear model (LM) analyses of bulk sets of genes, models were fitted to log_2_ transformed FPKM data and statistics extracted using the *lm* and associated functions in *R*. For analyses focussing on smaller numbers of genes, equivalent models were run with transformations applied on a case-by-case basis based on standard model diagnostics. Small structural equations models (path analysis) and generalized additive models (GAMs) were respectively implemented in the R packages *Lavaan* and *mgcv*. All analyses with *R* used version 3.1.0.

### Availability of supporting data

Sequencing data will be available in the European Nucleotide Archive under primary accession number PRJEB13319. Other supporting data are available as additional files.

## Additional files

Additional file 1: Table S1. Based on genome-wide (RNAseq) expression data, here we provide lists of annotated winter-biased and summer-biased genes, as defined by synchronous season-specific expression across study sites. (XLS 593 kb) Additional file 2: Table S2. Based on genome-wide (RNAseq) expression data, here we provide lists of seasonally-biased KEGG and REACTOME gene sets as determined by Gene Set Enrichment Analysis (GSEA). (XLS 64 kb) Additional file 3: Table S3. Details of the selected gene sets used to probe winter- and summer-biased gene lists and/or gene modules from network analyses. (XLS 98 kb) Additional file 4: Figures S1-S2. Supplementary analysis: body length as an age indicator, based on fishes monitored in outside artificial habitats, and potential age variation in study samples. (PDF 138 kb) Additional file 5: Figure S3. Supplementary analysis of confounder-adjusted seasonal effects on genome-wide (RNAseq) expression data. (PDF 405 kb) Additional file 6: Table S4. Gene Set Enrichment Analysis (GSEA) of RNAseq data with genes ranked by confounder-adjusted seasonal expression. (XLS 65 kb) Additional file 7: Table S5. List of core seasonally-biased immune genes determined from RNAseq data. To populate this list, seasonally-biased *ImmPort* list genes were individually assessed for their immunological significance. (XLSX 20 kb) Additional file 8: Table S6. Characteristics of fishes used for transcriptomic and tissue-specific analyses. (PDF 200 kb)

## Abbreviations

APC: antigen presenting cell
FDR: false discovery rate
FPKM: fragments per kilobase of exon per million fragments mapped
FRN: llyn Frongoch
GAM: generalized additive model
GSEA: gene set enrichment analysis
ILT: interbranchial lymphoid tissue
LM: general linear model
MHC: major histocompatibility complex
PCA: principal components analysis
PCO: principal co-ordinates analysis
Q-PCR: quantitative real-time PCR
RHD: afon Rheidol
SEM: structural equation model
STO: river Stour (Sussex)
TLR: toll-like receptor
UTC: co-ordinated universal time
