# Supplementary material for: Seasonal immunoregulation in a naturally-occurring vertebrate
Source: BMC Genomics. 2016 May 18;17:369. doi: 10.1186/s12864-016-2701-7 (PMC4870750; doi:10.1186/s12864-016-2701-7)
Supplement: Additional file 4: Figures S1-S2. — Supplementary analysis: body length as an age indicator, based on fishes monitored in outside artificial habitats, and potential age variation in study samples. (PDF 138 kb) [file 12864_2016_2701_MOESM4_ESM.pdf]

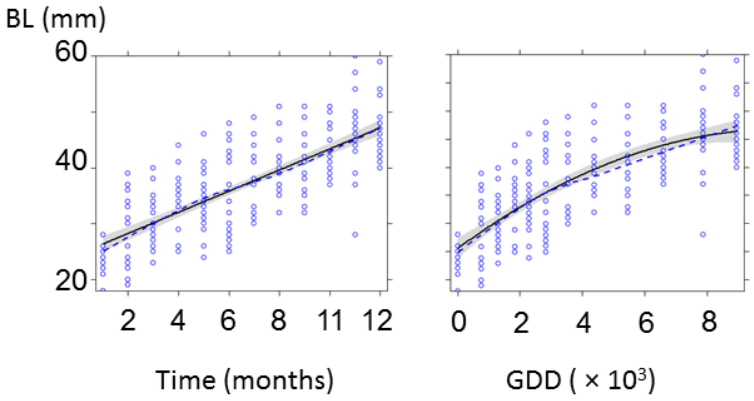

Fig. S1. Body length (BL) as an age indicator in fishes monitored in outdoors artificial habitats. Data represent single (end-point) measurements for 239 individual fishes maintained in 12 replicate outdoors artificial habitats, with 12 × monthly sampling points (see Methods). Fish varied in length at the start of the experiment (by a factor of up to × 1.6), having been captured as early juveniles of unknown age in the wild and stocked in the artificial habitats 4-6 weeks prior to the period of observation. Despite this initial heterogeneity, both time and growing degree days (GDD, see below) predicted 57% of the variation in body length during the period of observation. A linear regression was the best model for time (left panel) and a quadratic regression for GDD (right panel); this difference may be because, in the case of the linear regression on time, deceleration of growth rate in older/larger individuals may be counter-balanced by acceleration of growth at warmer temperatures. Plots show scatter of individual data and predicted effects with 95% confidence band (grey) and a loess smoother (blue dashed).

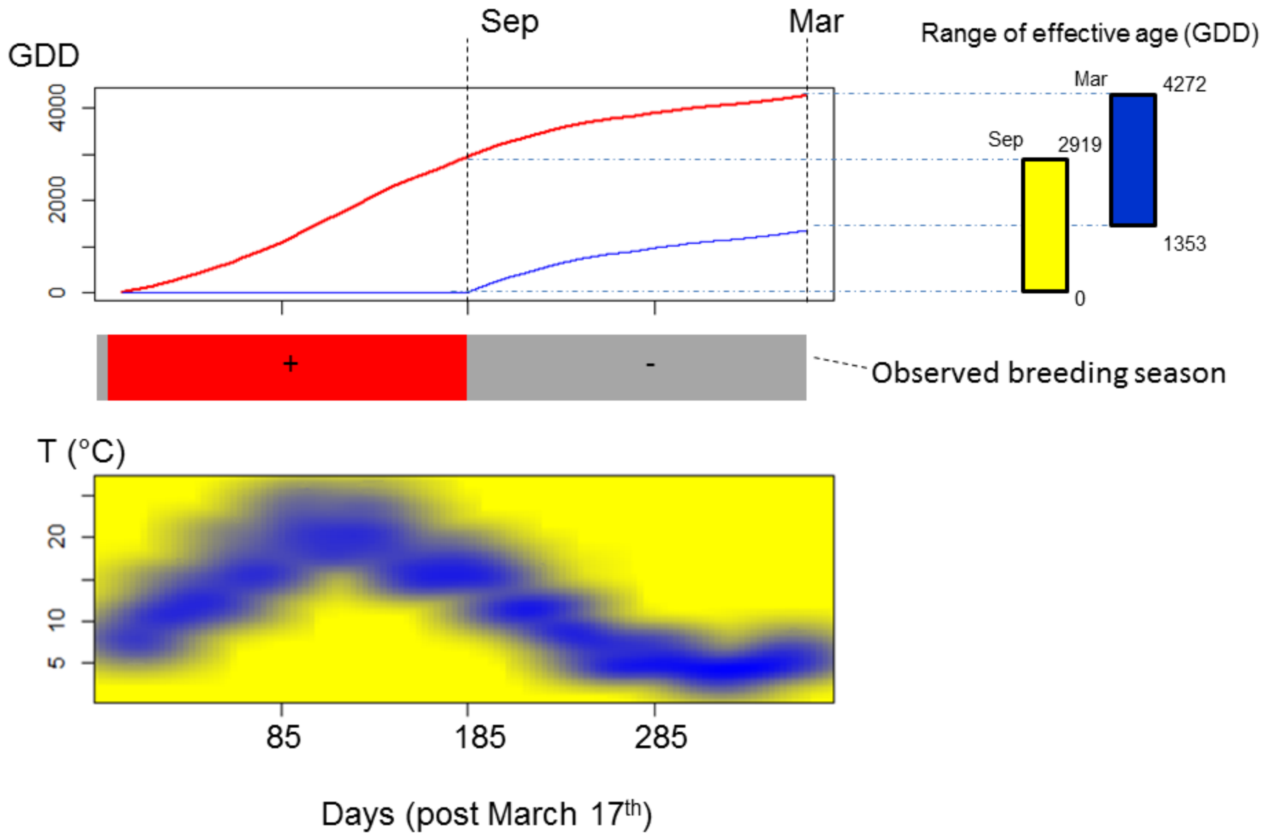

Fig. S2. Effective (biological) age considered as a function of growing degree days (GDD), and possible effective age ranges in September and March samples. At RHD, FRN and artificial habitats stocked from FRN, we observed some animals in breeding condition (males in breeding colouration or females with ripe ovaries) in every month from March to September (middle panel, above). Timing the respective onset and cessation of reproduction (fertilization) from the 17th of these months, and using field temperature records at FRN in 2014-2015 as a representative example, this would give a 0^+ cohort with a range of effective ages (GDD) of 0-2919 at a September 17th sampling point and 1353-4272 at a March 17th sampling point. However, it is likely that two year cohorts were present in our transcriptomic samples leading to even greater overlap in effective age (in this example 0-7191 GDD at the September sampling point and 1353-8544 at the March sampling point if 0^+ and 1^+ cohorts were present). Thus it is likely there was extensive overlap in effective age between the September and March sampling points in our study, which could be adjusted for in statistical models using body length as a surrogate for age (see Fig. S1). GDD were calculated using the mean daily temperature (based on measurements at 5 minute intervals) and a base of 0^oC.

Lower panel shows a smoothed colour density representation of temperature measurements (recorded at 5 minute intervals) at FRN during 2014-2015 (darker blue indicates a higher density of measurements). Based upon these measurements, upper panel shows a plot of the effective age, in GDD, of 0^+ individuals produced at the start (red) and end (blue) of the annual reproductive window. Bars indicating the range of effective

ages for the 0^+ cohort at September (yellow) and March (blue) sampling points are shown to the right.
