# Supplementary material for: Seasonal immunoregulation in a naturally-occurring vertebrate
Source: BMC Genomics. 2016 May 18;17:369. doi: 10.1186/s12864-016-2701-7 (PMC4870750; doi:10.1186/s12864-016-2701-7)
Supplement: Additional file 5: Figure S3. — Supplementary analysis of confounder-adjusted seasonal effects on genome-wide (RNAseq) expression data. (PDF 405 kb) [file 12864_2016_2701_MOESM5_ESM.pdf]

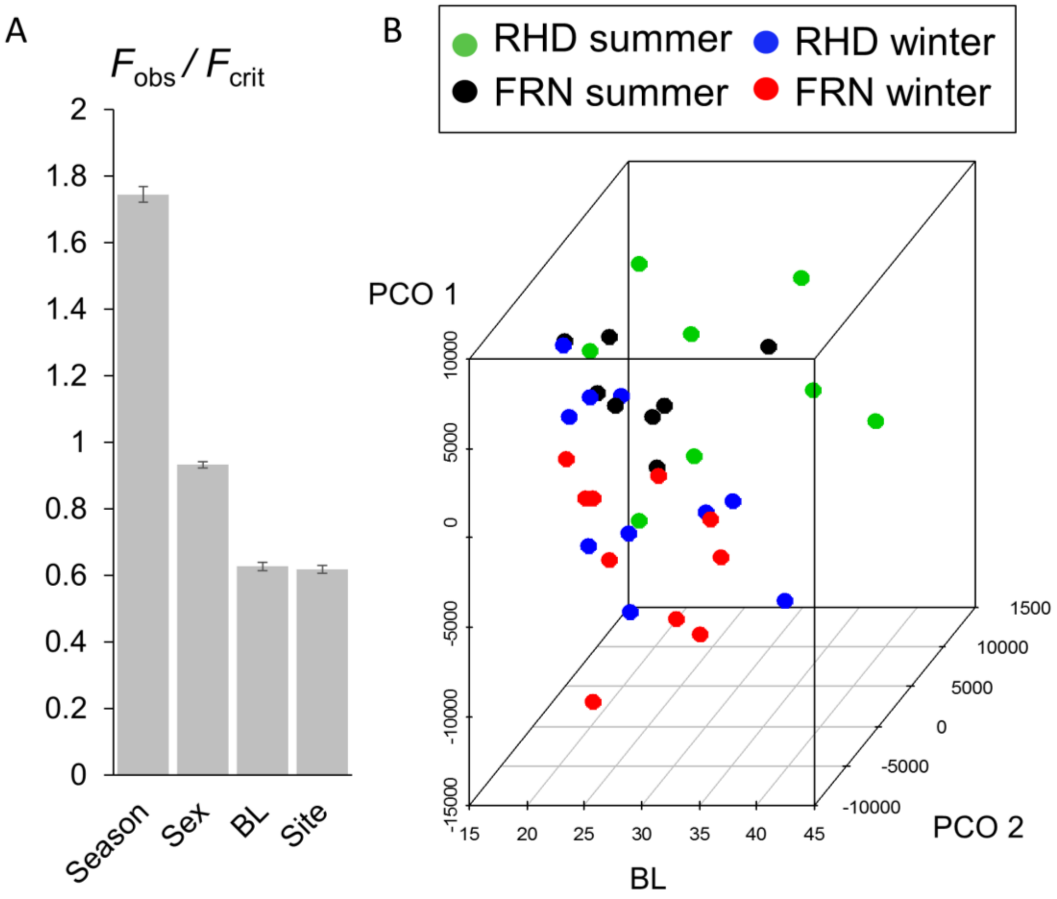

Fig. S3. Supplementary analysis of confounder-adjusted seasonal effects on genome-wide (RNAseq) expression data. (a) To each in turn of the 11455 genes used in Gene Set Enrichment Analyses (GSEA) we applied general linear models (LMs) of the form: expression ~ season + sex + body length (BL) + site. The bar chart shows summary statistics for *F* values from the LMs, where the observed *F* values (F_{obs}) are expressed as a proportion of the critical value (F_{crit}); bars are the mean ± 1 SE. Season was the main source of variation overall. (b) We also carried out a principal co-ordinates analysis (PCO) of the gene expression variables in the GSEA dataset. This demonstrated strong differentiation by season but no differentiation by length, sex or site. Variation along the two major axes of the analysis was significant for season (axis 1, P = 0.003; axis 2, P = 0.0004), but not for other terms, in LMs with the same explanatory terms as above. The 3-d plot shows co-ordinate scatter for individual fishes against the 2 major axes of the PCO and against length.
