# Supplementary material for: Seasonal immunoregulation in a naturally-occurring vertebrate
Source: BMC Genomics. 2016 May 18;17:369. doi: 10.1186/s12864-016-2701-7 (PMC4870750; doi:10.1186/s12864-016-2701-7)
Supplement: Additional file 8: Table S6. — Characteristics of fishes used for transcriptomic and tissue-specific analyses. (PDF 200 kb) [file 12864_2016_2701_MOESM8_ESM.pdf]

Characteristics of fishes used for transcriptomic and tissue-specific analyses

Site	Season	Body length (mm)	Sex (M:F)
FRN	Summer	20-29	4:4
FRN	Winter	20-32	7:3
RHD	Summer	19-39	3:5
RHD	Winter	19-41	3:7
STO	Summer	24-35	2:3
STO	Winter	26-49	4:6
